# Demographic history of Canary Islands male gene-pool: replacement of native lineages by European

**DOI:** 10.1186/1471-2148-9-181

**Published:** 2009-08-03

**Authors:** Rosa Fregel, Verónica Gomes, Leonor Gusmão, Ana M González, Vicente M Cabrera, António Amorim, Jose M Larruga

**Affiliations:** 1 Department of Genetics, University of La Laguna, Avda. Astrofísico Fco. Sánchez, La Laguna, 38271 Santa Cruz de Tenerife, Spain; 2 Institute of Pathology and Molecular Immunology of the University of Porto (IPATIMUP), University of Porto, Porto, Portugal; 3 Institute of Legal Medicine, University of Santiago de Compostela, Santiago de Compostela, Spain

## Abstract

**Background:**

The origin and prevalence of the prehispanic settlers of the Canary Islands has attracted great multidisciplinary interest. However, direct ancient DNA genetic studies on indigenous and historical 17^th^–18^th ^century remains, using mitochondrial DNA as a female marker, have only recently been possible. In the present work, the analysis of Y-chromosome polymorphisms in the same samples, has shed light on the way the European colonization affected male and female Canary Island indigenous genetic pools, from the conquest to present-day times.

**Results:**

Autochthonous (E-M81) and prominent (E-M78 and J-M267) Berber Y-chromosome lineages were detected in the indigenous remains, confirming a North West African origin for their ancestors which confirms previous mitochondrial DNA results. However, in contrast with their female lineages, which have survived in the present-day population since the conquest with only a moderate decline, the male indigenous lineages have dropped constantly being substituted by European lineages. Male and female sub-Saharan African genetic inputs were also detected in the Canary population, but their frequencies were higher during the 17^th^–18^th ^centuries than today.

**Conclusion:**

The European colonization of the Canary Islands introduced a strong sex-biased change in the indigenous population in such a way that indigenous female lineages survived in the extant population in a significantly higher proportion than their male counterparts.

## Background

The Canary Islands are a volcanic archipelago consisting of seven main islands situated in the Atlantic Ocean, facing the western Saharan coast of Africa. Fuerteventura and Lanzarote are the easternmost islands, the former being only a hundred km from the continent.

The Islands were already known to Mediterranean Classical cultures, but the Archipelago was rediscovered and visited by Genovese, Majorcan, Portuguese and French sailors during the 13^th ^and 14^th ^centuries. Under the auspices of the Castilian crown, Europeans conquered the Canary Islands during the 15^th ^century, beginning with Lanzarote in 1402 and finishing with Tenerife in 1496. The conquest was rather violent because the Guanches often fought fiercely against the invaders. Even islands such as Lanzarote or Gomera, which pacifically received the first Norman and Castilian expeditions, were the scene of violent revolts because the natives were enslaved in large numbers to defray the cost of the military expeditions. In retaliation, the rebels, mainly men, were killed and massively deported by the conquerors [1].

There are several questions about the past and present of the Guanches that have attracted the curiosity of scientists since the 19^th ^century. They refer to the time(s) and way(s) they arrived on the islands, their geographic origin, and whether their descendants persist in the present-day population [2]. The oldest human settlement seems to be no earlier than the first millennium B.C., according to absolute C^14 ^dating [3]. Coalescence age estimates obtained from mitochondrial DNA (mtDNA) [4] and Y-chromosome [5] putative founder lineages concord with archeological results.

As the islands were never connected with the African Continent, they had to be reached by sea. Their inhabitants did not supposedly have seafaring skills and communication among islands was thus absent at the time of the Spanish conquest. This poses the unresolved dilemma of whether the first settlers reached the islands by themselves and after that forgot their sailing skills or if they were transported to the islands by another maritime people [6].

From the beginning of the conquest, Guanche dialects and customs were found to be related to those of their N African Berber neighbors. Since then, anthropological, archeological and linguistic studies have provided further support to the N African origin of the indigenous population [7]. Furthermore, the different human types discovered and the heterogeneity of their cultural remains again points to the possibility of successive arrivals of N African settlers [8-13].

In spite of the aggressive conquest and subsequent massive European immigration and North and sub-Saharan African slave importation to the islands, historians estimated that approximately two-thirds of the Canary population were Africans and aborigines at the end of the 16^th ^century [14]. Moreover, osteological studies comparing aboriginal remains and modern rural populations, support the persistence of indigenous traits in the current population [10,15].

From the genetic perspective, strong evidence in support of a N African origin of the indigenous ancestors and their present-day persistence was only obtained when uniparental genetic markers were analyzed. Mitochondrial DNA (mtDNA) lineages, belonging to the U6 haplogroup [16], and Y-chromosome haplotypes of the E1b1b1b haplogroup, characterized by the M81 marker [17], both with a clear Berber origin, were detected in the Canary islanders at a significantly higher presence than in Iberians, their main colonizers [4,5]. In addition, admixture analysis taking the Iberians, Northwest and sub-Saharan West African populations as parental sources of the present-day Canary population, showed that the indigenous contribution was estimated to be 33% of maternal lineages [4] and only 7% for paternal lineages [5]. This strong sexual asymmetry was explained by a sociological bias favoring matings between Iberian males and indigenous females, and the greater indigenous male mortality during the Conquest [2]. Accordingly, intermediate admixture estimates were obtained when autosomal markers were used [18,19]. It is also worth mentioning that the detection of significant correlations between relative frequencies and/or diversity values for mtDNA, CD4/Alu haplotypes and ABO gene data, and geographical distances of the islands from Africa were explained assuming only one main colonization event [4,18,19]. On the contrary, using Y-chromosome markers, two opposite correlations were found [5], which was explained by at least two independent waves of colonists from NW Africa, still detectable today. These genetic results, although congruent with previous anthropological, archeological and linguistic data, have not been free of criticism. It is well known that admixture values strongly depend on the appropriate choice of the parental populations. To extrapolate the unknown indigenous population from a NW African sample pool seems unsuitable, because although the mtDNA haplogrup U6 present in the Canary Islanders and in North Africa originates in the latter [20], the most abundant Canary sublineage, U6b1, is absent in NW Africa, and the most abundant U6a sublineages on that continent are very scarce in the archipelago [4,16,21] pointing to different N African sources. Moreover, the unquestionably N African lineages present in the present-day Canary population may not be wholly due to the indigenous heritage but to Iberian colonizers, since these lineages, albeit in low frequencies, are also present in Spain and Portugal [22,23]. Another possibility is that those U6 lineages present in the islands may derive from slaves brought from the NW African coast after the conquest. However, all these concerns vanished when mtDNA information was obtained directly from indigenous remains [24], and exhumed 17^th^–18^th ^century remains from Tenerife [25]. The presence of U6b1 lineages and other presumed founder lineages were detected in both samples, confirming their prehispanic origin. In addition, the direct incorporation of the indigenous sample as a parental source of the admixed Canary Islands populations provided greater indigenous female component estimates (42–73%) than those based on the present-day NW African maternal gene pool (33–43%).

Although most of the populational molecular genetic studies carried out on skeletal remains have used mtDNA, mainly because of its copy number per cell, sex-typing based on the XY amelogenin test has also been frequently and successfully used since the beginning of the ancient DNA (aDNA) typing era [26-28]. Recent achievements in Neanderthal whole nuclear genome [29,30] and gene specific [31,32] studies prompted us to undertake a Y-chromosome SNP analysis in the indigenous population of the islands, which is crucial to determine the relative survival of the prehispanic male genetic pool in the present-day population. The goal was to directly type North-African geographically structured Y-chromosome binary markers in samples from indigenous and 17^th^–18^th ^century remains that were already successfully analyzed for mtDNA [24,25] and proven to be males by an amelogenin-based sexing test [33]. The statistical null hypotheses of these analyses would be that male haplogroup frequencies in the indigenous and historical samples should not be significantly different from those found in the modern Canary population.

## Results and discussion

### Sample typing and methods

First of all, contamination was not detected in extraction or PCR negative controls, in any case of Y-chromosome analysis, although sporadic contamination was observed when the previous mtDNA analysis was performed. PCR efficiency with the samples selected for the present Y-chromosome analysis was 58% in the indigenous material and 63% in La Concepción historical material. However, taking into account previous mtDNA and amelogenin analysis, only 30 (10%) of the total indigenous and 42 (21%) of the historical samples produced Y-chromosome positive results. From the 30 successfully amplified indigenous samples, 24 were from Gran Canaria, 3 from Fuerteventura, 2 from Tenerife and 2 from Gomera.

For the direct Y-chromosome markers amplification, a mean of 366 ± 254 initial molecules was quantified by real-time PCR. However, the limited amount of DNA substrate left after the mtDNA and amelogenin analysis, and the frequent PCR inhibition problems due to the relatively large amount of extract necessary to directly amplify each marker, required the inclusion of a prior preamplification step. At first, we unsuccessfully tried whole genome amplification. Secondly, we turned to a specific multiplex approach using the whole sixteen primer-pair set in one reaction but, although some specific products were obtained, the relative abundance of unspecific amplifications made this method difficult to apply. Only when the sixteen markers were subdivided into three different multiplex assays (Additional file 1), clean specific products were obtained in subsequent nested PCR reamplifications. Cloning and sequencing confirmed the PCR amplification specificity for all the markers used.

Although a hierarchical approach was followed in the RFLP analysis in La Laguna (Additional file 2), all the samples were first typed for the phylogenetically basal M89 marker, the three most frequent North African markers (M78, M81, M267) and M269, the most abundant European marker. The same five markers were also replicated in the Porto lab using a first multiplex SNaPshot analysis (Additional file 3). Samples derived for M78, M81, M267 and M269 were not included in further analyses; those derived for M89 were subsequently analyzed for the M9, M45, M170, M172, M173 and M201 multiplex set; and ancestral ones for the M2, M33, M34, M60 and M96 multiplex set.

### Authenticity of ancient DNA results

We are confident in the authenticity of our results for several reasons. First, only those samples that showed a relatively high initial copy number in the real-time PCR quantification assay were successfully analyzed. Second, we never detected contamination in any of the negative controls performed in extraction and amplification. Third, all the markers analyzed in the same individual always gave genealogically congruent results for their respective ancestral or derived status. Fourth, replication of all the samples in two independent laboratories produced identical results. Fifth, haplogroup types and frequencies obtained for the indigenous and historical samples were very different, but in accordance with the predictions based on historical and archeological records. Sixth, haplogroups crucial to the correct interpretation of the results, such as E-M81, were not detected in the panel of male researchers that handled the remains from each excavation (Additional file 4).

### Y-SNP haplogroups in indigenous and historical Canary Island populations

Y-SNP haplogroups in indigenous and historical Canary Island populations are shown in Table 1. The autochthonous N African E-M81 haplogroup was the most abundant type in the indigenous sample (26.7%). It is also the most common in NW Africa (64%) with its highest frequency in the Western Sahara (76%) [17,34]. The E-M81 marker is rare outside N Africa and its presence in the Iberian Peninsula has mainly been considered a result of Moorish influence [5,17]. In the historical sample, the E-M81 frequency was 11.9%, more similar to that found in the current Canary Islands (8.3%) than to the indigenous sample (26.7%). Taking into account the low frequency of this haplogroup in sub-Saharan Africa, its presence in the historical sample could be better explained by indigenous persistence than by later trade in sub-Saharan slaves. However, it is also to be expected that some E-M81 lineages reached the islands due to the minor NW African slave-trade. The notable E-M81 frequency decrease in the historical sample, relative to the indigenous one, is in agreement with a strong European replacement of the indigenous males at the beginning of the conquest [5].

**Table 1:** Y-chromosome haplogroup frequencies in the studied populations

**HG**	**MARKER**	**ABO**	**CON**	**HIE**^1^	**PAL**^1^	**GOM**^1^	**TFE**^1^	**GCA**^1^	**LAN**^1^	**FUE**^1^	**CAN**^1^	**NWA**^2,3^	**SAH**^2,3^	**NCA**^4^	**IBE**^5^
ADC	-														0.15

B	M60														

E*	M96						0.56				0.15	0.45			0.76

E1a*	M33	3.33	2.38						1.03	2.67	0.46	1.82	8.99	0.99	0.46

E1b1a*	M2		4.76	4.25	2.35			1.28	1.03		0.92	4.55	11.24	0.99	0.31

E1b1b1*	M35											4.09		2.97	

E1b1b1a*	M78	23.33	11.9	6.38	2.35	4.35	3.37	3.85	3.09	2.67	3.53	6.82		5.94	2.44

E1b1b1b*	M81	26.67	11.9	2.13	5.88	4.35	10.68	11.54	6.19	13.33	8.28	65.00	59.55	39.1	5.19

E1b1b1c1*	M34			2.13	3.53	2.17	3.93	2.56			2.30			2.97	1.68

F*	M89											0.91		6.44	0.31

G*	M201		2.38	4.25	2.35	5.44	3.93	3.85	5.16	2.67	3.99	0.91			4.27

I*	M170	6.67		2.13	9.41	20.65	7.30	6.41	13.40	5.33	9.66	0.45			9.77

J1*	M267	16.67	11.90	4.25	2.35	7.61	1.12	1.28	3.09	8.00	3.53	5.00	20.22	29.2	2.14

J2*	M172		2.38	14.89	14.12	10.87	7.30	7.69	12.37	10.67	10.43	4.09		3.47	7.02

K*	M9	10.00			4.71	1.09	6.18	5.13	1.03	1.33	3.37	1.82		0.99	3.21

P*	M45	3.33												0.50	0.46

R1a	M17		9.52	2.13	2.35	2.17	2.25	1.28	4.12	5.33	2.76			0.50	1.83

R1b1b2	M269	10.00	42.88	57.46	50.60	41.30	53.38	55.13	49.49	48.00	50.62	4.09		5.94	60.00

Sample		30	42	47	85	92	178	78	97	75	652	221	89	202	655

^1^Flores et al. 2001; ^2^Bosch et al. 2001; ^3^Flores 2001; ^4^Arredi et al. 2004; ^5^Flores et al. 2004

Comparison of Y-chromosome haplogroup frequencies (%) among the indigenous (ABO), historical (CON), and extant samples from the seven Canary Islands (Lanzarote: LAN; Fuerteventura: FUE; Gran Canaria: GCA; Tenerife: TFE; Gomera: GOM; La Palma: PAL; Hierro: HIE), total Canaries sample (CAN) and current Northwest African (NWA), North Central African (NCA), Saharan (SAH) and Iberian Peninsula (IBE) populations.

Congruently, the European R-M269 haplogroup was already the most frequent in the historical sample (42.9%). R-M269 reaches 60% in the Iberian peninsula [35] but is found at a low frequency in NW Africa (4–6%), and seems to have been introduced there from Europe in historical times [17]. Its frequency in the extant Canary population (53.2%) is similar to that found in the Iberian Peninsula, pointing to a mainly European origin for the present-day male pool in the Canaries [5]. The fact that a similar frequency has been found in the historical sample, again points to a strong European replacement of the male indigenous pool since the early conquest period. Surprisingly, R-M269 was also found in the indigenous sample in a moderate frequency (10%). Its presence in the indigenous people could be explained in two ways: (a) R-M269 was introduced into NW Africa in prehistoric not historical times, or (b) the presence of this marker in the aborigines was due to a prehispanic European gene flow into the indigenous population. As NW African R-M269 chromosomes showed close STR-similarity to the Iberian ones [17], pointing to recent contacts between both regions, the second option appears more plausible.

A sub-Saharan component is detected in both indigenous (3.3%) and historical (7.1%) samples. E-M33 was the only sub-Saharan marker found in aborigines. In Africa, its highest frequencies have been detected in Southern (51%) and Central areas (57%) [17,36]. However, as its frequencies in North-Central Moroccan Berbers (3.2%) and in Saharan people (3.5%) [34] are similar to that found in the indigenous sample, its prehispanic presence in the islands could be due to the same NW African colonization that brought E-M81. E-M33 was also detected in the historical population (2.4%) which, together with E-M81, could indicate a moderate indigenous Y-lineage persistence in the 17^th^–18^th ^centuries. Although its presence could also be the result of the later sub-Saharan slave trade, its limited frequency in the Gulf of Guinea [17], the main source of slaves, makes this second option less probable. The E-M2 branch is another sub-Saharan haplogroup [37,38] present in the historical sample (4.76%). It reaches its highest frequency in Mali and has been proposed as a marker of the Bantu expansion [38]. So, its presence in the 17^th^–18^th ^century population could indicate direct influence due to slavery. In fact, it is well documented that, for instance, in Gran Canaria more than 10,000 slaves were introduced during the 16^th ^century. The majority of these slaves came from regions of sub-Saharan Africa [39] where E-M2 is the most abundant Y-chromosome haplogroup [17,36]. E-M2 is also present in NW African populations [17,34] so, although this marker was not detected in our small indigenous sample, a prehispanic NW African origin cannot be ruled out.

Some additional haplogroups detected in indigenous and/or historical Canaries samples (M78, M172, M173, M201 and M267), appear in the Iberian Peninsula as well as in NW Africa [17,34-36,40-47]. Nevertheless, M78 and M267, which are more abundant in the latter (Table 1), have a higher frequency in the indigenous sample (23.3% and 16.7%, respectively) than in the 17^th^–18^th ^centuries population (11.9% in both cases), which is again in accordance with a NW African origin for the prehispanic colonizers of the islands.

Due to the low variance of J-M267 in N Africa compared to that in the Middle East, its presence in the former has been related to the Arab expansion in the 7^th ^century A.D. [36]. However, if the arrival of the indigenous people in the islands was around 1,000 years B.C. [48], the presence of J-M267 in NW Africa could be previous to the Arab expansion. Alternatively, this marker might have reached the islands with a second wave of colonists.

Similarly to E-M81, the frequencies of E-M78 and J-M267 decrease in the historical and present-day Canary populations, again highlighting the strong demic impact of the European colonists before the 17^th^–18^th ^centuries. On the other hand, haplogroups with a comparatively higher European presence such as M172, M201 and M173 (comprising SRY1532 and M17) were only detected in the historical sample, therefore, they most probably reached the islands after the European conquest.

The presence of the I-M170 haplogroup in the indigenous sample (6.7%) deserves special attention. This haplogroup is the only major clade of the Y-chromosome phylogeny that is widespread over Europe and almost absent elsewhere, suggesting that it originated there [49]. It is especially abundant in the eastern Mediterranean area, with its highest frequencies in the Balkans [50]. Therefore, the presence of this European Y-chromosome lineage in the indigenous pool is compatible with a direct Mediterranean input, or to a more ancient demic influx from Europe to N Africa than has yet been proposed [17].

### Genetic distances and AMOVA

**Figure 1:**
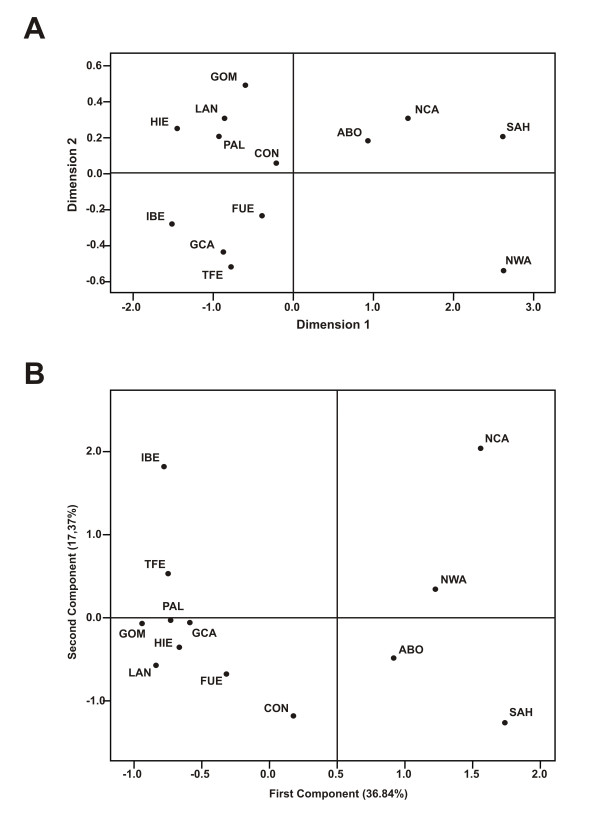
**MDS and PCA graphical representations**. A) MDS plot based on pairwise Fst genetic distances. B) PCA results based on haplogroup frequencies. Population codes are as in Table 1.

In order to detect genetic differences between populations, pairwise F_ST _comparisons (Table 2) were carried out. It was found that the indigenous Canary Island population has its highest affinities with N Central Africa (p = 0.01) and with the historical population (p = 0.002), compared to the rest of the samples (p < 0.0001). In turn, the historical sample was more closely related to the present-day Canary populations (from p = 0.43 to p = 0.02) than to the Iberian Peninsula (p = 0.003), being highly divergent from Africa (p < 0.0001). These relative relationships are graphically represented in the bidimensional plot of the multidimensional scaling (MDS) analysis performed with the F_ST _distance matrix (Figure 1a). The indigenous sample is halfway between N Central Africa and the 17^th^–18^th ^century sample; the latter standing closer to the present-day Canary populations and to the Iberian Peninsula. Results from the principal component analysis (PCA) are highly congruent with the MDS plot (Figure 1b). The only discrepancy is that, in this case, the indigenous sample is closer to NW Africa than to N Central Africa. The first principal component (accounting for 37% of the whole variance) clearly separates Canarian aborigines and Africans from the present-day Canary and Iberian samples, leaving the historical sample in an intermediate position. Haplogroups E-M81 and J-M267 on the one hand, and R-M269, G-M201 and J-M172 on the other, are mainly responsible for these positive and negative displacements. Additionally, in the second component (17% of the whole variance) the sub-Saharan haplogroup E-M96 is the main source of the positive displacements of the Iberian peninsula and N Central Africa from the present-day Canary Islanders and the group composed by NW African, indigenous and Saharan populations, respectively. On the negative side, the sub-Saharan E-M2 and E-M33 haplogroups clearly make the Eastern islands of Fuerteventura and Lanzarote closer to the historical sample, and the aborigines to the Sahara (Figure 1b).

**Table 2:** F_ST _distances between populations based on Y-chromosome haplogroup frequencies

	**ABO**	**CON**	**FUE**	**LAN**	**GCA**	**TFE**	**GOM**	**HIE**	**PAL**	**NWA**	**SAH**	**NCA**	**IBE**
**ABO**	-												

**CON**	0.083**	-											

**FUE**	0.135***	0.000	-										

**LAN**	0.177***	0.021*	0.000	-									

**GCA**	0.188***	0.018	0.000	0.001	-								

**TFE**	0.187***	0.022*	0.002	0.003	0.000	-							

**GOM**	0.133***	0.030*	0.014	0.000	0.024*	0.023**	-						

**HIE**	0.233***	0.022	0.005	0.002	0.000	0.005	0.029*	-					

**PAL**	0.181***	0.023*	0.000	0.000	0.000	0.000	0.008	0.000	-				

**NWA**	0.160***	0.363***	0.383***	0.470***	0.463***	0.443***	0.441***	0.588***	0.480***	-			

**SAH**	0.140***	0.329***	0.364***	0.457***	0.468***	0.449***	0.409***	0.579***	0.469***	0.032***	-		

**NCA**	0.040*	0.158***	0.196***	0.264***	0.273***	0.273***	0.223***	0.319***	0.269***	0.096***	0.050***	-	

**IBE**	0.285***	0.046**	0.017*	0.008	0.000	0.004	0.035***	0.003	0.006	0.565***	0.583***	0.381***	-

*p < 0.05; **p < 0.01; ***p < 0.001

Codes as in Table 1.

AMOVA analyses were performed to assess the relative amount of variance attributed to differences among and within natural geographic areas. When the indigenous and historical samples were included within the Canarian group, 80% of the total variance was observed within populations (F_ST_), 3% among populations within groups (F_SC_) and 17% among groups (F_CT_). However, when the indigenous sample was removed from the analysis or grouped with N Africa, F_CT _increased to 18%, whereas F_SC _decreases to 2.4%. When, in addition to the aborigines, the historical sample was also removed, the variance partition values did not change. These results indicate, once more, that the indigenous sample is comparatively more similar to the N African than to the present-day Canary population, while the C 17^th^–18^th ^historical sample shows more affinities with the modern Canary Island population.

### Male vs. female contributions

To explain the current demographic composition of the Canaries, in previous genetic approaches their present-day inhabitants were considered as a hybrid population with a NW African substrate, contributed most probably by Berber indigenous founders, a substantial European input and, to a lesser degree, a sub-Saharan African component, introduced after the conquest as slaves. Consequently, admixture estimates were calculated using present-day Iberian, NW African and sub-Saharan W African populations as parental sources. Results based on uniparental markers have provided contradictory evidence pointing to a considerable sexually asymmetric contribution, with a predominant (92%) male European contribution [5] and a high (33–43%) NW African female component [4,51]. More recently, the maternal indigenous substrate has been directly estimated from ancient remains [24] showing a higher indigenous contribution (42–73%) than that estimated when the present-day NW African mtDNA gene pool was used. Admixture proportions based on mtDNA were also calculated for the 17^th^–18^th ^century population of Tenerife [25] and a higher sub-Saharan African influence was found (14%) than in the present-day population (5%).

The Y-chromosome data obtained in the present study, from the same indigenous and historical populations, allowed a similar direct analysis of the male gene-pool (Table 3). Iberian males appear as the main contributors to the extant Canary population (83.0% ± 4.7%) but to a lesser extent than the indirect estimation (92%). Accordingly, the indigenous component (16.1% ± 4.6%) is also higher than before (7%), whereas the sub-Saharan input was similar (1%). When these indigenous and sub-Saharan male contributions were compared to their respective female contributions, a significant sex bias favoring indigenous (p < 0.01) and sub-Saharan (p < 0.05) female persistence was observed. As for the 17^th^–18^th ^century historical population, although the main contribution was already Iberian (63.2 ± 14.5%), at that time the indigenous (31.1 ± 14.0) and sub-Saharan (5.8 ± 4.4) influences were greater than today but, in this case, male and female contribution comparisons did not reach significant differences. Nevertheless, these results have to be taken with caution because the historical sample has been taken from a single burial in Tenerife, whereas the indigenous sample is made up of several archeological sites from different islands.

**Table 3:** Admixture estimations

		**Iberian peninsula**	**Aborigines**	**Sub-Saharan Africa**
**Canarian males**				
	17th–18th centuries	63.2 ± 14.5	31.1 ± 14.0	5.8 ± 4.5
	Present day	83.0 ± 4.7	16.1 ± 4.6	0.9 ± 0.7

**Canarian females**				
	17th–18th centuries	47.9 ± 23.3	39.9 ± 22.9	12.2 ± 6.5
	Present day	55.4 ± 15.6	41.8 ± 15.8	2.8 ± 3.7

Relative indigenous, Iberian and West sub-Saharan African contributions to the 17th–18th century and present-day Canary Islands populations, were estimated based on Y-chromosome and mtDNA haplogroup frequencies in the three paternal populations.

## Conclusion

The presence of autochthonous North African E-M81 lineages, and also other relatively abundant markers (E-M78 and J-M267) from the same region in the indigenous population, strongly points to that area as the most probable origin of the Guanche ancestors. This is in accordance with previous genetic studies performed on the same material at mtDNA level [24], and in support of the cultural connections found between the Berbers and the indigenous islanders people [9,15,52]. In addition to this mainly NW African colonization, the detection in the indigenous sample of markers like I-M170 and R-M269 of clear European ascription might suggest that other secondary waves also reached the Archipelago, most likely from the Mediterranean basin. This would again be in agreement with the multiple settlement theory proposed to explain the physical and cultural diversity found between and within the different islands [3,52]. However, as these markers are also present in N Africa, albeit in low frequencies, it could be that they arrived in the islands during the same African wave(s) that brought E-M81 and reached relatively high frequencies there due to founder and genetic-drift effects. If so, the presence of these markers in N Africa may be older than previously proposed [17].

Compared to the original natives, the 17^th^–18^th ^century historical sample mainly differs by harboring lower frequencies of NW African haplogroups (p < 0.05), such as E-M81 (11.9% vs 26.7%), E-M78 (11.9% vs 23.3%) and J-M267 (11.9% vs 16.7%), and higher frequencies for European haplogroups (p < 0.001) like R-M269 (42.9% vs 10.0%) or R-M173, (9.5% vs 0.0%). A notable exception was I-M170 because it was not detected in the historical sample, despite being moderately frequent in the aborigines (6.7%).

Different founder effects on different islands could be a plausible explanation, since all the natives carrying I-M170 were from Gran Canaria, whereas the historical sample was taken from Tenerife. Another difference between these two samples is the higher, albeit not significant, frequency of sub-Saharan lineages (7.1% vs 3.3%) in this historical population. However, these differences were not detected at mtDNA level [24,25], as the NW African haplogroup U6 (10.2% vs 10.0%) and the most abundant and widespread European haplogroup H (46.9% vs 52.1%) showed similar frequencies in both samples. The sharp and swift change observed for the indigenous male and female genetic pools can be satisfactorily explained if it is accepted that indigenous females were reproductively more successful after the conquest than males, who were displaced by male European colonizers. Although sampling bias and drift effects could also explain these differences, the genetic data corroborate historical chronicles that narrated frequent mass killings and deportations of mainly males during the conquest [1,9]. Even after that first violent period, the better social and economical position held by the Europeans continued to favor their mating with indigenous females.

The asymmetric sexual evolution of the mixed population is also corroborated when quantitative admixture estimates are independently applied to their female and male genetic pools at different times (Table 3). The Iberian contribution to the male genetic pool increases from 63% in the 17^th^–18^th ^centuries to 83% in the present-day population, which is accompanied by a parallel dropping of the male indigenous (31% vs 17%) and sub-Saharan (6% vs 1%) contributions. However, relative proportions in the female pool are strikingly constant for Iberians (48% vs 55%) and aborigines (40% vs 42%), from the 17^th^–18^th ^centuries to the present [53], and only the sub-Saharan female contribution shows an important decrease (12% vs 3%).

These results indicate that indigenous males were negatively discriminated, not only at the beginning of the conquest but also afterwards. In the case of the sub-Saharan lineages, it seems that their mating disadvantage affected both sexes, although more so in males.

It has been stated that the Canary Islands served as a laboratory for the later conquest and settlement of the American Continent by the Spaniards [54,55]. In fact, recent genetic studies on Iberoamerican populations [56-58] have also detected considerable sexual asymmetry, showing that the European male contribution to their present-day genetic pools is significantly greater than the female, as happens in the Canary Islands. Ironically, autochthonous male M81 and female U6 lineages from the Canaries have also been detected in Iberoamerica [57], demonstrating that Canary Islanders with indigenous ancestors actively participated in the American colonization.

## Methods

### Samples

Samples used in this study were excavated by different authorized archeological teams. The material ceded to perform molecular analyses consisted, in all cases, of teeth without fractures. Whenever possible, teeth were directly taken from their mandible alveolus. A total of 643 teeth corresponding to 493 different individuals were analyzed. This material belonged to different indigenous burials sampled from six of the seven islands: Fuerteventura (13 teeth from 10 individuals), Gran Canaria (230 teeth from 115 individuals), Tenerife (45 teeth from 39 individuals), Gomera (62 teeth from 52 individuals), Hierro (44 teeth from 44 individuals) and La Palma (43 teeth from 38 individuals). Calibrated radiocarbon dating was performed in the Beta Analytic Radiocarbon Dating Laboratory (Miami). At least two samples for site were analyzed. Aboriginal remains were clearly pre-conquest for all the analyzed islands: Tenerife (2210 ± 60 to 1720 ± 60 BP), Gomera (1743 ± 40 to 1493 ± 40 BP), Hierro (1740 ± 50 to 970 ± 50 BP) and Gran Canaria (1410 ± 60 to 750 ± 60 BP) [33]. Although the Fuerteventura and La Palma [59] materials were not directly C-14 dated, ceramic types co-excavated with the remains indicate that they were also prehispanic and not older than 1000 years BP. Historical remains, from 17^th^–18^th ^century, exhumed from La Concepción Church in Tenerife (206 teeth from 195 individuals), were also analyzed. In order to avoid sampling repetitions, individuals from different graves were preferably chosen, and only one type of tooth was taken when more than one individual was sampled in the same grave [25].

### Ancient DNA laboratory

To ensure the reliability of the results, strict measures were taken to avoid contamination, as recommended for aDNA work [60,61]. Analyses were performed in three independent aDNA-dedicated laboratories. In the first, the excavated material was decontaminated and processed to obtain powdered samples. In the second, DNA extraction and pre-PCR procedures were carried out. PCR amplifications were performed in a third area. Finally, post-PCR analyses were done in another physically isolated laboratory.

In each aDNA dedicated area, all personnel were required to wear lab-coats, face-shields, hats and multiple pairs of gloves. The equipment and work areas were constantly irradiated with UV lamps and frequently cleaned with bleach. All sample manipulations were performed in laminar flow cabinets, with dedicated pipettes and sterile filter tips (Tip One, Star Lab). Solutions were commercially acquired whenever possible; otherwise, they were autoclaved and UV-treated. All metallic material was sterilized in an oven at 200°C for at least 4 h.

### Ancient DNA extraction

Initial decontamination steps were carried out on all samples prior to extraction. Teeth were thoroughly washed with 15% HCl, rinsed with UV-treated ddH_2_O and exposed to UV light for 10 min. In order to reconstruct teeth after extractions, they were transversely cut through the mid-line, using a dental electric saw, and the internal pulp and dentine drilled out using a dental drill. The powder was collected in 1.5 ml sterile tubes and DNA extracted according to a modified GuSCN-silica based protocol [24,25,62].

### Previous mtDNA and amelogenin analysis

As it there are estimated to be about 3,000 mtDNA molecules per cell [63], previous to the Y-chromosome study all the teeth were analyzed for mtDNA [24]. Those individuals that could not be amplified for mtDNA (35%) were not included in subsequent analysis. The successfully amplified mtDNA samples were sexed using an amelogenin test as previously published [25,33]. For those samples carrying the Y-chromosome specific band, two additional amelogenin typings were performed to confirm the result. When only the female band was amplified, 4 to 5 additional repetitions were carried out, in order to avoid false results due to allelic dropout during the first few PCR cycles [33]. In the indigenous sample, only 49% of the individuals were unequivocally sexed and 17% proved to be male, so 89 teeth from 52 individuals (14.6%) were analyzed for Y-chromosome binary markers. For the historical sample, 56% of the individuals gave results for the amelogenin locus and 34% resulted male, so 67 individuals were included in the Y-chromosome analysis.

### Y-SNP selection

**Figure 2:**
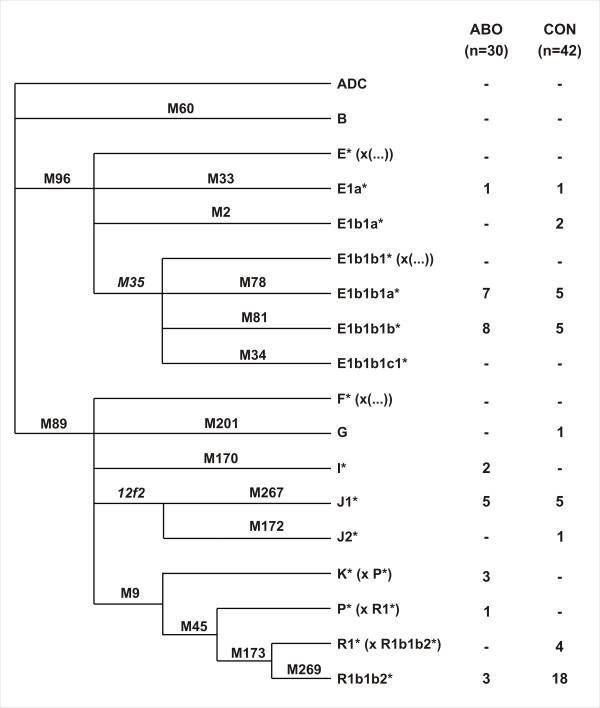
**Y-chromosome tree of haplogroups and absolute frequencies for each population**. Y-chromosome tree, taken from Karafet et al. (2008), representing the genealogical relationships of the haplogroups characterized in this study, using diagnostic SNPs and their absolute frequencies in the indigenous (ABO) and historical (CON) samples from the Canaries.

Sixteen biallelic markers (M2, M9, M33, M34, M45, M60, M78, M81, M89, M96, M170, M172, M173, M201, M267, M269; see Figure 2), that characterize the most prevalent lineages in NW Africa, Sub-Saharan Africa and Europe, were chosen from the literature [5,17,34-36,38,40]. The Y-SNP haplogroup nomenclature and tree topology, represented in Figure 2, were established following the nomenclature of Karafet et al. 2008 [64].

To amplify the Y-SNPs, primers were designed to define fragments with less than 100 base pairs (bp), as recommended for aDNA studies, using Primer3 software [65]. Different sets of primers were used for: a) direct SNP amplification, b) primer-extension preamplificaction (PEP), c) nested-PCR reamplification and d) SNaPshot multiplex SNP typing. Primer sequences are shown in Additional files 1, 2 &3.

### Real-time PCR quantification

To assess the number of molecules used as template for PCR amplification [66], we used iQ™ SYBR^® ^Green Supermix (BioRad) in an iCycler Thermal Cycler (BioRad). Primers and thermal cycling conditions were as described for Y-SNP amplifications. Tenfold serial dilutions of a purified and quantified standard were included in the experiments to determine the standard curve in order to estimate the initial number of DNA molecules in each sample.

### Primer-Extension Preamplification (PEP)

Primer-extension preamplification of the whole genome [67] was carried out using DOP PCR Master Mix Kit (Roche) and following the manufacturer's protocol.

### Multiplex preamplification

Multiplex amplification was performed in two different ways. In the first approach, the thirty-two primers of the sixteen markers, detailed in Additional file 1, were used in a PCR multiplex. Subsequently, the sixteen markers were amplified in three different multiplex assays (Additional file 1). PCR was performed in 10 μl volume, containing 1 μl of 10× Tris-HCl buffer, 200 μM of each dNTP, 5 mM of MgCl_2_, 1.5 ng of bovine serum albumin (BSA), 1 unit of Taq polymerase (Ecogen), the optimal concentration for each pair of primers (Additional file 1) and 5 μl of DNA extract. When no amplification product was obtained, the DNA extract volume was increased to 7 μl in subsequent PCRs. To overcome PCR inhibition, detectable by the lack of primer-dimers, DNA was reduced to 3 μl and/or the Taq and BSA amounts were doubled. Reactions were submitted to 40 amplification cycles with denaturation at 94°C for 10 s, annealing at 55°C for 10 s and extension at 72°C for 10 s. Extraction and PCR controls were included to detect modern DNA contamination. Ancient female DNA was used as an additional negative control.

### Nested-PCR reamplification

Each marker was reamplified using a nested-PCR approach. In these amplifications, one of the previous PCR primers was used together with a newly designed nested-primer (Additional file 2). The PCR was run in 40 μl, containing 4 μl of 10× Tris-HCl buffer, 200 μM of each dNTP, 40 pmoles of each primer, 5 mM of MgCl_2_, 3 units of Taq polymerase (Ecogen) and 8 μl of 1:200 diluted multiplex PCR product. Reactions were submitted to 40 amplification cycles with denaturation at 94°C for 10 s, annealing at 55°C for 10 s and extension at 72°C for 10 s. A 5 μl aliquot of the PCR product was loaded in 10% acrylamide:bis-acrylamide (19:1) gels, stained with ethidium bromide and visualized under UV to assess the amplification yield.

### RFLP analysis

0.5–1 unit of the appropriate restriction enzyme (Additional file 2) was used to directly digest 10 μl of the nested-PCR product under the manufacturers' recommendations. RFLP patterns were resolved on 8% acrylamide:bis-acrylamide (19:1) in 1× TBE buffer and stained with ethidium bromide (1 μg/ml) for 15 min.

### Multiplex SNaPshot analysis

Products of each nested PCR were pooled in 0.5-ml sterile eppendorf tubes at comparatively optimal amounts, and ethanol precipitated in order to purify and concentrate the samples in a 10 μl volume. In order to remove any primers and dNTPs left by the previous ethanol precipitation, 1 μl of the concentrated PCR products was treated with 0.5 μl of Exo-SAP-it (USB) and incubated at 37°C for 15 min, followed by heating at 85°C for 15 min to inactivate the enzyme.

The multiplex minisequencing reactions were carried out in a 5 μl final volume containing 1 μl of SNaPshot™ Multiplex Ready Mix (Applied Biosystem) and 1.5 μl of the previously treated PCR products. Concentrations of primers in the reaction mix are specified in Additional file 3. Reactions were submitted to 25 cycles of denaturation at 96°C for 10 s, annealing at 50°C for 5 s, and extension at 60°C for 30 s. Final extension products were treated with 1 μl of SAP (USB) and incubated at 37°C for 1 h, followed by enzyme inactivation by heating at 85°C for 15 min.

For capillary electrophoresis, 0.5 μl purified extension products were mixed with 9 μl Hi-Di™ formamide (Applied Biosystems, (AB)) and 0.5 μl of internal size standard GeneScan-120 LIZ™ (AB). Samples were run on an ABI PRISM 3130 Genetic Analyzer (AB) using POP-7^® ^(AB). Results were analyzed using GeneMapper 4.0 software (AB).

### Cloning and sequencing

To check the specificity of the primers, PCR products of each marker were ligated into pGEM-T vectors (Promega). Colonies were plated on selective Amp/IPTG/X-gal plates, and white colonies were selected. Clones were directly sequenced using M13 universal primers. Sequencing reactions were prepared in 10 μl volumes using the BigDye 3.1 Terminator Cycle Sequencing kit (AB) and the products were run on an ABI PRISM 310 Genetic Analyzer (AB).

### Contamination prevention and authentication

To avoid modern contaminant DNA during the mtDNA and amelogenin analyses, all the previously reported procedures were followed [24,25,33,62]. In addition, for the Y-chromosome analysis, aDNA was exclusively manipulated by female researchers and all sample analyses were duplicated, in La Laguna, using RFLP assays and in Porto, using SNaPshot analysis.

### RFLP analysis on modern populations

In order to make comparisons among populations feasible, after concluding the analysis of all the extant aDNA samples from the Canary Islands [5], samples from the Iberian peninsula [35] and North Africa [68] were additionally typed for M269 marker as previously described [69].

### Statistical analyses

The indigenous and historical samples were compared between each other and with each present-day island population, with West Saharan (including Mauritanian samples), NW African (comprising Arabs and Berbers from Morocco) and North Central African populations (including Algerian and Tunisian samples), and with an overall sample from the Iberian Peninsula as detailed in Additional file 5. To make comparisons possible, frequencies were calculated for haplogroups at the same level of SNP resolution as the indigenous and historical samples. Analysis of molecular variance (AMOVA) and pairwise F_ST _genetic distances based on haplogroup frequencies [70] were performed using ARLEQUIN 2000 package [71]. Principal component (PC) and multidimensional scaling (MDS) analyses were carried out using the SPSS statistical package 11.5 (SPSS, Inc). Admixture analysis, using Y-chromosome SNPs (k = 18) as alleles of a single locus, was performed using ADMIX 2.0 program [72]. Admixture coefficients and their standard deviations were obtain from 3000 bootstrap replicates. Contingency and Fisher exact tests were used to assess the significance of haplogroup frequency differences. To test the significance of admixture proportions between male and female lineages, we used a significance test of independent proportions [73].

## Authors' contributions

The experiments were designed by all the authors. RF and AMG carried out the RFLP analyses, VG and LG the SNaPshot analyses, while VMC and JML analyzed extant samples for M269. All the authors participated in data analysis, discussion of results and drafting the manuscript. All authors read and approved the final manuscript.

## Supplementary Material

Additional file 1 **PCR conditions**. Primer sequences, optimal concentrations and product lenghs for the Multiplex PCR assays Click here for file

Additional file 2 **PCR and RFLP conditions**. Primer sequences for reamplification PCRs and RFLP assay patterns Click here for file

Additional file 3 **SNaPshot conditions**. Single Base Extension (SBE) primer sequences and optimal concentrations for SNaPshot analysis Click here for file

Additional file 4 **Y-chromosome haplogroup data**. Y-chromosome haplogroups for male researchers involved in the study Click here for file

Additional file 5 **Sample size and references for populations used in this study**. Populations used in analysis Click here for file
